# VISIBILITY OF PROGRAMS AND SERVICES ON HOSPITAL WEBSITES: GERIATRICS COMPARED WITH OBSTETRICS

**DOI:** 10.1093/geroni/igz038.1675

**Published:** 2019-11-08

**Authors:** Brenda P Johnson, MyoungJin Kim, Emily E McMahon, Ali Mojadam

**Affiliations:** Illinois State University, Normal, Illinois, United States

## Abstract

Hospitals increasingly use a digital platform to market their services as well as reflect the values and mission of the organization. As payment incentives and national initiatives grow for making hospitals more “age friendly” in both the quality and extent of specialized services for older adults, the visibility of these services on a digital platform is of interest. This exploratory, descriptive analysis of hospital websites was to illuminate the ease with which a consumer could identify that a hospital has specialized services in geriatrics as compared with obstetrics. A proportionate stratified random sampling based on the five geographical regions of the country was used to select 220 hospitals with 5% margin of error, 95% confidence interval and 50% sample proportion with no prior information from a population of over 600 hospitals known to have implemented at least one quality initiative specific to older adults (NICHE). A binary matrix was developed for collecting data related to the visibility of services in three main areas of hospital websites: 1) images and terms on the homepage 2) identification of programs in the “Services” function and 3) availability of the specialty in the “Search for Doctor” function. Data show that the frequency of all key words used to identify obstetrical services was greater than those used to describe geriatric services across all functional areas explored in this study. Implications are that hospital websites may be an underutilized resource for marketing services as well as accurately reflecting specialized services for the older adult population.

